# Multiple asystole events in a patient undergoing total knee arthroplasty - a case report

**DOI:** 10.1186/s12871-019-0777-8

**Published:** 2019-06-14

**Authors:** Anna-Maria Burgdorff, Lilit Flöther, David Wohlrab

**Affiliations:** 1 Department of Anesthesiology and Surgical Intensive Care Medicine, University Hospital Halle (Saale), Ernst-Grube-Straße 40, 06120 Halle (Saale), Germany; 2 Department of Orthopedic Surgery and Traumatology, University Hospital Halle (Saale), Halle (Saale), Germany

**Keywords:** Cardiac arrest, Bradycardia, Asystole, Total knee arthroplasty

## Abstract

**Background:**

Unexpected cardiac arrest in patients during surgery is associated with high mortality. Reasons are often multifactorial and unclear.

**Case presentation:**

This case report describes a patient who developed reversible asystole during knee surgery under general anaesthesia. All diagnostic cardiac examinations were unremarkable. After surgery, the patient showed no further symptoms.

**Conclusion:**

To prevent cardiac arrest due to non-cardiac reasons, patients with a high risk for asystole caused by vasovagal reflex or by pain need to be identified. Preoperative conditions such as hypovolemia need to be improved prior to surgery, and additional monitoring should be used. Further investigations to determine the influence of non-cardiac disease and long-term medication are necessary.

## Background

Cardiac arrest in patients undergoing surgery is often dramatic. Mortality rates of over 50% have been documented. The overall incidence of perioperative cardiac arrests is estimated to be between 4.3 and 34.6 per 10,000 procedures, which rises to 54.4 per 10,000 in the elderly population [1]. In total knee arthroplasty (TKA), Berstock et al. (2018) reported a 90-day mortality of 0.39% (out of 700,981 TKAs from 1991 to 2014) [2].

The reasons for cardiac arrest are frequently multifactorial, depending on the preoperative conditions of the patient, their cardiac risks, anaesthesia management, human factors and the surgical procedure itself [1]. Specific risk factors for cardiac events in TKA are prior cardiac events, chronic hypertension, diabetes mellitus, increased age and male sex. Some factors were shown to be protective, such as being overweight (BMI between 26 and 30 kg/m^2^) [2, 3].

One reason for cardiac arrest could be an overexcitation of the parasympathetic nerve system. Pain, for example, could lead to bradycardia followed by asystole, as in cases of vasovagal syncope or the Bezold-Jarisch reflex [4].

The following case report describes reversible asystole events during knee arthroplasty under general anaesthesia.

## Case presentation

We present the case of a 71-year-old self-employed, non-smoking German female patient scheduled to undergo a right TKA. Relevant past medical history included type 2 diabetes mellitus treated with insulin (HbA1c 43 mmol/mol), BMI of 35.5 kg/m^2^ (176 cm/ 110 kg), arterial hypertension (usual value 140/60 mmHg via right arm) and restless leg syndrome. Important self-medications were metformin, valsartan, hydrochlorothiazide, nebivolol, aspirin, lercanidipine hydrochloride, levodopa and benserazide hydrochloride. The patient’s history included a TKA on the right side in 2000, a traumatic dislocation in 2011 and a revision arthroplasty in 2012 due to instability. These operations were performed under general anaesthesia without complications.

In February 2018, the patient presented to our orthopaedic outpatient department because of increasing pain in the right knee joint. Examinations showed implant loosening and *Staphylococcus epidermidis* infection. Therefore, the patient was scheduled for a two-stage revision with implant removal and antibiotic-loaded spacer implantation. Antibiotic therapy was deliberately withheld in view of the patient’s stable, non-septic clinical parameters and to better evaluate potential antibiotic sensitivities following surgical removal of the infected prosthesis. For pain management, she received a prescription for celecoxib and metamizole per os (PO), as well as subcutaneous antithrombotic prophylaxis with enoxaparin sodium. The patient was advised to follow the rest/ice/compression/elevation (RICE) protocol during the time until surgery.

In the premedication visit, the patient was classified as ASA III (according to the American Society of Anaesthesiologists) with a metabolic equivalent of ≥4. An electrocardiogram (ECG) and current lab values (erythrocytes 7.0 mmol/l; Hb 7.0 mmol/l; Hk 0.32 l/l; CRP 5.7 mg/l; all others were without abnormalities) had already been determined. The patient showed no signs of cardiopulmonary decompensation such as dyspnoea, oedema or auscultatory abnormalities at the time. On the day of surgery, 5 mg oxazepam PO and the patient’s usual medication, except metformin, valsartan and hydrochlorothiazide, were administered. The patient was informed that she could eat until 6 h before surgery and drink clear liquids until 2 h before surgery. The procedure was performed under general anaesthesia with endotracheal intubation. The initial vital parameters were a blood pressure (BP) of 160/80 mmHg and a heart rate (HR) of 65 bpm. Anaesthesia induction was performed under standard monitoring (non-invasive BP, HR and pulse oximetry) in the following order: propofol (180 mg) intravenous (IV), sufentanil (20 μg) IV and rocuronium (50 mg) IV. Sevoflurane (target value of minimal alveolar concentration of 0.8–0.9) and sufentanil (10 μg as a single IV bolus at the incision site) were used to maintain anaesthesia.

Furthermore, 1 g of tranexamic acid IV and balanced electrolyte solution (2 l of Jonosteril, which includes sodium chloride, sodium acetate trihydrate, potassium acetate, magnesium acetate tetrahydrate, calcium acetate) was given during the entire surgery. After anaesthesia induction, BP was 95/55 mmHg, and BP measurement was performed every 3 minand documented every 5 min. Because of a fall in blood pressure, the patient received norepinephrine (20 ml/h at 3 μg/min via IV perfusion) for 20 min to increase the mean arterial pressure to 65 mmHg. This regimen was performed directly after anaesthesia induction and was stopped at when the mean arterial pressure reached 65 mmHg, which occurred after 28 min (after the initial surgical incision). Thereafter, the patient was stable without catecholamines.

After incision and preparation of the knee, the surgeon observed pronounced synovitis and intramedullary femoral and tibial periprosthetic infected membranes. A smear was collected, and the patient received cefuroxime at 1.5 g IV. During the tibial component removal, spontaneously resolving episodes of asystole were observed on 3-lead-ECG. The asystole events were observed twice over a maximum of 2 s and depended on the surgical manipulation. Before any intervention, the events ended spontaneously with complete removal, and no haemodynamic changes were observed. Because of the short action and no haemodynamic changes, as well as the observation of stable blood pressure, we checked the 3-lead-ECG to exclude an artefact due to the manipulation and the high sensibility of the derivation. The operation proceeded without abnormalities until 45 min after incision, and the surgeon began the intramedullary reaming. This procedure led to a seven-second asystole again and was associated with a fast fall in BP (42/18 mmHg), oxygen saturation (68%) and end-tidal CO_2_ (21 mmHg). We informed the surgeon and interrupted the intervention. Epinephrine was prepared but not injected because at the same time, the asystole vanished. There was a complete recovery of haemodynamic parameters (92/56 mmHg; 98%; 42 mmHg). The heart rate was 52 bpm. The patient received 0.5 mg of atropine IV to prevent reproducible asystole for the rest of the procedure. Catecholamines were not necessary because of increased BP. Other reasons for asystole were checked, without any indication of a reversible cause. It is possible that the manipulations (reaming) led to pain with a vasovagal reaction, but around these events, the patient had no signs of pain, such as hypertension or tachycardia. From the advent of asystole until the end of the surgery, the depth of anaesthesia was monitored by the bispectral index (BIS), with no evidence of low anaesthesia (BIS score of 42) after the last event. According to our standard operation procedure, BIS was not indicated for this surgery, but we wanted to ensure an adequate depth of anaesthesia while minimising sevoflurane anaesthetic administration due to the patient’s comorbidities until the end of operation.

Extubating was performed without any problems. The patient received metamizole (1 g) at the end of surgery to prevent postoperative pain. In the recovery room, the patient first received a 12-channel ECG, which was without any abnormalities. Laboratory tests and blood gas analysis (troponin, BNP, CK-MB, D-Dimers, electrolytes) were performed to exclude ischaemia, embolic events, infarction or changes in electrolytes as the reason for asystole. We performed a case conference with our cardiologists. The subsequent transthoracic echocardiography was also without any abnormalities according to the age of our patient. Non-invasive cardiovascular investigations, such as repeated 12-channel-ECG, 24-h Holter monitoring and ultrasound of extracranial vessels, were performed. These investigations revealed minor supraventricular ectopic activity but were otherwise unremarkable. The cardiologist assumed that the patient had a vagal reaction when bone manipulations were performed by the surgeon and advised atropine IV (without recommendation of a dose) for the following operations.

Six weeks later, the patient underwent scheduled spacer removal and TKA. The patient received atropine IV (1 mg) after induction of anaesthesia to reach a higher HR and underwent invasive BP measurement and BIS monitoring. As the surgeon manipulated the medullary cavity, the patient developed a self-limiting episode of bradycardia (40 bpm) lasting only 3 s. No other events were recorded during surgery or hospital stay.

## Discussion and conclusions

The patient had intraoperative asystole events during manipulation of the medullary cavity without an apparent primary cardiac cause.

First, the pre-existing conditions of our patient are known as risk factors for cardiac adverse events. With respect to studies about mortality in TKA, hypertension requiring medication is one of the predictors for cardiac complications in TKAs (OR 4.74; 95% CI 1.04 to 21.59; *p* = 0.0440) [5], as well as type 2 diabetes mellitus treated with insulin (OR 1.95; 95% CI 1.13 to 3.35; *p* = 0.016) [6].

In addition, the literature describes that periodic leg movements in patients with restless legs syndrome (RLS) during sleep produce episodes of tachycardia followed by bradycardia. This has been reported less frequently in the elderly, with women having a higher prevalence of bradycardia during these episodes [7, 8]. At the time of medullary manipulation and asystole, there was no measurement of relaxation or BIS monitoring. We can only guess the patient was in the same state as sleep. Cholley-Roulleau et al. (2017) [8] found no association between RLS and cardiovascular diseases; however, this is not conclusively clarified in the general neurological literature.

In addition to the pre-existing conditions of the patient, another cause for the asystole events could be a relative imbalance between the para- and sympathetic nervous systems, resulting in a decreased HR, venous pooling and loss of vascular tone, which would be consistent with the findings of our cardiologists. This proposed pathway agrees with the pathophysiology of the Bezold-Jarisch reflex. A vasovagal syncope as a cause for an asystole is also conceivable. Pain stimuli and decreased venous return, transmitted by the glossopharyngeal and vagal nerves, perhaps led to activation of the medullary vasomotor centre. Increased vagal activity stimulates the parasympathetic system accompanied by depression of sympathetic activity, followed by bradycardia, vasodilation and decreased release of catecholamines [4]. In addition, the surgical positioning (supine position and intermittently lifting of the right leg) and procedure, accompanied by the patient’s pre-existing state of severe obesity, leads to an increased intrabdominal pressure in the patient, resulting in compression of the inferior vena cava with reduced venous return and lower right arterial pressure. The patient also did not wear compression stocking on the left non-operated leg, which may have aggravated venous pooling. Regarding reduced venous return and possible higher risk for vasovagal reactions, haemoglobin levels should also be considered. The patient had a haemoglobin level of 7 mmol/l at the beginning of surgery. The haemoglobin level fell to 5.9 mmol/l during surgery. Under catecholamine therapy, the mean arterial pressure of the patient was more than 60 mmHg with normal HR, even if the systolic BP of the patient was lower than that during her everyday life. According to the criteria of patient blood management (PBM) [9, 10], there was no indication for a blood transfusion. The patient received 2 l of balanced electrolyte solution as volume replacement. Blood loss (approximately 800 ml) could cause lower venous return, resulting in an enhanced vasovagal reaction. Irrespective of the recommendations of the PBM criteria, it is conceivable that the patient would have needed higher blood levels of haemoglobin, which was not noticeable because of the effects of the usual medication (e.g., no tachycardia due to beta blocker therapy, no changes in BP after stabilisation with catecholamine treatment). Regarding the infection of the prothesis and the re-operation, a higher volume of blood loss should be considered, and in preparation for surgery, anaemia should have been treated in this case. There are current guidelines for PBM, especially for patients undergoing knee and hip arthroplasty [11, 12]. In our patient, the ferritin level should have been determined, and if necessary, iron and/or erythropoietin could have been replaced [11, 12]. Spahn (2010) [13] described significantly increased mortality in patients with pre- and postoperative anaemia undergoing total hip or knee arthroplasty. It is possible that this patient group needs a blood transfusion earlier to improve postoperative outcomes.

With respect to the anaemia and reasons for an imbalance between the para- and sympathetic nervous systems, the usual medication should also be considered. Therefore, long-term ACE inhibitor and a diuretic agent were not administered the day of surgery because of the increased risk for intraoperative hypotension through hypovolemia and due to pre-existing anaemia, which would lower compensatory mechanisms via the renin angiotensin system, followed by failed vasoconstriction and enhanced Bezold-Jarisch reflex [14]. It is possible that similar effects can be observed in RLS. Dopamine, a natural catecholamine, showed reduced effects in our patient because of the mentioned lower dopaminergic striatal receptor binding and reduced compensatory pathways.

With regard to a vasovagal hypothesis and after the recommendation of our cardiologist, we changed our procedure for the following surgery of the patient. Before re-implantation of the prosthesis, we injected atropine (1 mg) IV at the beginning of anaesthesia to reduce the reaction of the vagal nerve. Additionally, the patient underwent intraarterial BP monitoring (highest value 145/60 mmHg and lowest value 100/50 mmHg) for better control of volume therapy (pressure pulse variation) and faster reactions for changes in BP. The exclude pain due to a low level of anaesthesia as well as to deep anaesthesia as a reason for asystole and activation of the Bezold-Jarisch reflex, we used BIS monitoring (between 37 to 60). Repeated blood gas analyses were performed to control blood loss (Hb/Hk), volume therapy (lactate) and electrolyte changes. Haemoglobin was 7.0 mmol/l the day before surgery and changed during the operation to 5.5 mmol/l, which was followed by postoperative blood transfusion. The patient was stable except for during the short-term bradycardia. In comparison to the first operation, there were no differences, besides a higher dosage of sufentanil (35 μg for induction + 10 μg). This higher dosage was based on the values of BIS monitoring (from 42 to 60) and BP as signs of pain (increased from 100/50 mmHg and over 120/65 to 140/70 mmHg when ending catecholamine treatment). The monitoring of deep anaesthesia and volume therapy as the main reasons for vasovagal reaction, which led to higher drug doses and transfusion during the implantation surgery, could as well as increase the patient’s HR due to atropine, which may explain the better outcome after the second surgery.

Limitations of our intraoperative care during the first surgery should be mentioned as well. Normally, anaemia due to iron deficiency should be screened and treated. Because of the infection, there was not enough time to wait and treat the patient over weeks with PO drugs, but preoperative screening via labour and IV supplementation of iron could have been performed. Another point could be an earlier supply of invasive blood pressure measurement before the beginning of surgery or after the first asystole to see the changes more clearly and to allow blood gas analyses. We also changed the 3-lead-ECG to a 5-lead ECG intraoperatively to obtain better information about the changes in heart rhythms. There was no reason from the beginning of surgery to use BIS or 5-lead-ECG.

The cause of asystole in our patient seemed to be multifactorial. To prevent such adverse events during further operations in patients similar to this patient, some points should be noted. Pre-existing conditions, such as hypovolemia and anaemia, need special attention and should be treated before surgery. Patients with risks for Bezold-Jarisch reflex or other vasovagal reactions (anaemia, hypovolemia, receiving an operation with high pain, vena cava compression due the patient’s positioning, cardiac interventions, lower preload, etc.) must be evaluated before surgery, and prophylactic measurements should be performed (for example, BIS, blood gas analysis, etc.). Therefore, patients with prothesis infection, hypovolemia risk and higher intraoperative bleeding risk should have invasive blood pressure measurements applied and dynamic parameters available for volume therapy, such as pressure pulse variation, passive leg rising tests and blood gas analyses. To exclude pain or to reduce the risk for pain, additional peripheral regional anaesthesia in patients with chronic pain, change in prosthesis or repeated interventions are recommended.

## Data Availability

The datasets generated and analysed for the case report are not publicly available due to protect participant confidentiality. They are available from the corresponding author on reasonable request.

## Abbreviations

BIS: Bispectral index
BP: Blood pressure
ECG: electrocardiogram
HR: Heart rate
IV: intravenous
PBM: Patient blood management
PO: per os
RLS: Restless legs syndrome
TKA: Total knee arthroplasty
